# Molecular Detection of Antibiotic-Resistant Genes in *Pseudomonas aeruginosa* from Nonclinical Environment: Public Health Implications in Mthatha, Eastern Cape Province, South Africa

**DOI:** 10.1155/2021/8861074

**Published:** 2021-01-05

**Authors:** Mojisola Clara Hosu, Sandeep Vasaikar, Grace Emily Okuthe, Teke Apalata

**Affiliations:** ^1^Division of Medical Microbiology, Department of Laboratory Medicine and Pathology, Faculty of Health Sciences, Walter Sisulu University, Private Bag: X1, Mthatha 5117, Eastern Cape, South Africa; ^2^National Health Laboratory Services (NHLS), Nelson Mandela Academic Hospital, Mthatha 5100, South Africa; ^3^Department of Biological and Environmental Sciences, Walter Sisulu University, Private Bag, X1, Mthatha 5117, Eastern Cape, South Africa

## Abstract

Evaluation of resistant profiles and detection of antimicrobial-resistant genes of bacterial pathogens in the nonclinical milieu is imperative to assess the probable risk of dissemination of resistant genes in the environment. This paper sought to identify antibiotic-resistant genes in *Pseudomonas aeruginosa* from nonclinical sources in Mthatha, Eastern Cape, and evaluate its public health implications. Samples collected from abattoir wastewater and aquatic environment were processed by membrane filtration and cultured on CHROMagarTM *Pseudomonas* medium. Species identification was performed by autoSCAN-4 (Dade Behring Inc., IL). Molecular characterization of the isolates was confirmed using real-time polymerase chain reaction (rPCR) and selected isolates were further screened for the possibility of harboring antimicrobial resistance genes. Fifty-one *Pseudomonas* species were recovered from abattoir wastewater and surface water samples, out of which thirty-six strains were *Pseudomonas aeruginosa* (70.6%). The *P. aeruginosa* isolates demonstrated resistance to aztreonam (86.1%), ceftazidime (63.9%), piperacillin (58.3%), cefepime (55.6%), imipenem (50%), piperacillin/tazobactam (47.2%), meropenem (41.7%), and levofloxacin (30.6%). Twenty out of thirty-six *P. aeruginosa* displayed multidrug resistance profiles and were classified as multidrug-resistant (MDR) (55.6%). Most of the bacterial isolates exhibited a high Multiple Antibiotic Resistance (MAR) Index ranging from 0.08 to 0.69 with a mean MAR index of 0.38. In the rPCR analysis of fifteen *P*. *aeruginosa* isolates, 14 isolates (93.3%) were detected harboring *bla*_SHV_, six isolates (40%) harbored *bla*_TEM_, and three isolates (20%) harbored *bla*_CTX-M,_ being the least occurring ESBL. Results of the current study revealed that *P. aeruginosa* isolates recovered from nonclinical milieu are resistant to frontline clinically relevant antipseudomonal drugs. This is concerning as it poses a risk to the environment and constitutes a public health threat. Given the public health relevance, the paper recommends monitoring of multidrug-resistant pathogens in effluent environments.

## 1. Introduction

Antimicrobial resistance (AMR) is a public health crisis in both human and veterinary medicine [1, 2]. The irrational use of antibiotics in both human medicine and animal production for growth-promoting purposes, metaphylaxis, and prophylaxis has fueled the proliferation and spread of antibiotic-resistant bacteria and resistance genes resulting in aggravated public health and environmental risks [3–5]. The threat posed by AMR to human health is particularly concerning in low- to middle-income countries (LMICs). This is due to the higher possibility of community-acquired resistant infections, the high transmissible disease burden in the general populace, and poor access to health services [6], thereby leading to increased morbidity, prolonged hospitalization, and increased healthcare costs, thus exerting an economic burden on family units and the society [7].

*Pseudomonas aeruginosa* (*P. aeruginosa*), an environmental bacterium, can be found in various terrestrial and aquatic habitats. This is due to its extensive metabolic versatility that enhances its distribution, proliferation, and survival despite adverse physical and chemical conditions, thereby enhancing its ecological success and potential threat to public health [8, 9]. *P. aeruginosa* is an opportunistic human pathogen competent for a wide array of infections including respiratory tract, blood, urinary tract, and skin infections [10]. This competence for infections and ability for antibiotic resistance has made the organism to be recognized as a threat to public health [9, 10]. *P. aeruginosa* make use of both intrinsic and acquired resistance mechanisms. The acquired resistance mechanism is facilitated by mobile genetic elements through horizontal gene transfer (HGT). This poses a greater risk to human health because of the ease of expression and dissemination [11].

Antibiotic usage in the agricultural sector has compounded the spread of resistance in the human community due to the environmental dissemination of transferable resistance genes [12]. Abattoir wastes have the ability to contaminate both surface and groundwater. Discharge from abattoir effluent contaminates the environment by introducing pathogens that can affect land and water qualities, thus endangering human, animal, and aquatic ecosystem's health and constituting a menace to human health and environmental safety [13]. The possibility of pathogens from abattoir effluent and animal waste reaching or discharging into water bodies and developing resistance to antibiotics in human infection is a concern. This is because these infections are usually difficult to treat and often result in morbidity and mortality especially in the most vulnerable members of the community [14].

Several studies have investigated the prevalence and detection of extended-spectrum *β*-lactamase- (ESBL-) and metallo *β*-lactamase- (MBL-) producing *P. aeruginosa* isolated from clinical samples [15, 16], but there is a scarcity of data on the occurrence in nonclinical samples. The need to identify and monitor antibiotic-resistant genes in water bodies and wastewater is necessary to assess their potential risk to human health. The present study investigated the prevalence of antimicrobial resistance and antibiotic-resistant genes in nonclinical strains of *Pseudomonas aeruginosa* in Mthatha, Eastern Cape, and highlighted its public health implications.

## 2. Materials and Methods

### 2.1. Study Design and Setting

A cross-sectional study was conducted within the period of January to June 2019. The study sites were Umzikantu Red Meat Abattoir, Zimbane Mthatha, Mthatha River, and Mthatha Dam. Umzikantu Red Meat Abattoir is a high-throughput abattoir located in Zimbane location in Mthatha, Eastern Cape. It is the only operational red meat abattoir serving Mthatha and its environs in the OR Tambo District Municipality. It is certified and has the capacity to slaughter 50 units of animals on a daily basis. One unit equals one cow/ox or two calves or six sheep or four pigs. The abattoir is open to the public and offers slaughter and cutting services at an affordable price. It also doubles as a wholesaler supplying meat to butcheries, supermarkets, and hospitals.

Mthatha Dam (31°33′2″S 28°44′24″E Coordinates) is an earth-fill type dam on the Mthatha River, located close to Mthatha Town, in the OR Tambo District Municipality of the Eastern Cape. This dam was built in 1977 to serve municipal and industrial purposes. The Department of Water and Sanitation oversees the affairs of the dam. The catchment area of the dam is 886 km^2^ with a surface area measuring 25.42 km^2^. It has a height of 38 m with a length measuring 620 m. The reservoir capacity of the dam is 253,674,000 m^3^.

### 2.2. Sample Collection

Abattoir wastewater samples: using standard methods for the examination of water and wastewater [17], 100 mL of abattoir effluents was taken from two sampling points into sterile bottles appropriately labeled. All samples were stored in cooler boxes for transportation to the Medical Microbiology laboratory at Walter Sisulu University Mthatha, for further analyses, within 4 h of sample collection.

Aquatic environment samples: water samples from the Mthatha Dam were collected aseptically in sterile 100 mL Duran Schott glass bottles from different sampling points by directly dipping the bottles about 20 cm below the surface of the water. After collection, the samples were stored in iced cooler boxes, transported to the laboratory, and kept at about 4°C until analyzed.

### 2.3. Bacteriological Analysis

The membrane filtration method was used for isolation according to standard methods [17]. For all samples, three volumes of 100 mL were filtered [10] through a 0.45 *μ*m pore sized gridded membrane filter (Whatman Laboratory Division, Maidstone, England) using a water pump (model Sartorius 16824). Filters were removed and aseptically placed on CHROMagarTM *Pseudomonas* (CHROMagarTM; Paris, France) agar plates ensuring that no air bubbles were trapped. All media were prepared according to the manufacturers' instructions (CHROMagarTM; Paris, France). Each sample was analyzed in triplicate. The plates were incubated aerobically at 37°C for 24–48 hours. Blue colonies which were characteristics of *Pseudomonas* spp. were subcultured to obtain pure cultures.

### 2.4. Characterization of Pseudomonas aeruginosa

Blue colonies typical of *Pseudomonas* species on the chromogenic medium were subcultured on both Cetrimide agar and CHROMagar to get pure colonies. The characteristic grape-like odor was a useful marker of identification. Phenotypic tests such as Gram stain, oxidase test, and catalase test were performed [18]. Species identification was carried out using Gram-negative ID type 2 panels (Beckman Coulter, Inc. USA) of MicroScan autoScan-4 automated system (Dade Behring Inc., Deerfield, IL). Growth at 42°C [18] in an aerobic incubator was also used to confirm the identity of the *P. aeruginosa* isolates. All of the strains were stored at ‒80°C in 15% glycerol until further use.

### 2.5. Molecular Confirmation of Strains by rPCR

DNA Extraction: DNA was extracted from overnight colonies of a bacterial culture grown on Cetrimide agar. This was resuspended in Roche MagNA Pure Bacteria Lysis Buffer, vortexed briefly, heated at 95°C for 10 minutes, and pelleted by centrifugation at 13000 *g* for 10 minutes. Four hundred microliters were used as a specimen in the MagNA Pure Compact (MPC) System (Roche Applied Science, Indianapolis), using MPC Nucleic Acid isolation kit 1 according to the manufacturer's instructions. Elution tubes containing 200 *µ*l purified nucleic acids were stored at ‒80°C until further use. The LightCycler 2.0 instrument (Roche Applied Science, Germany) and Fast start LightCycler 480 HybProbes Master Kit (Roche Diagnostics, USA) were used for polymerase reaction. Specific primers and probes (Table 1) designed by TIB Molbiol (Germany) targeting the gene, species-specific *gyrB,* were amplified by singleplex real-time polymerase chain reaction (rPCR) following the protocol shown in Table 2.

### 2.6. Antimicrobial Susceptibility Testing

Antimicrobial susceptibility testing was performed by MicroScan autoScan-4 system using dehydrated broth microdilution method in the MIC Panel Type 44 (NM44) (Beckman Coulter, Inc. USA) following the manufacturer's guidelines [20]. The following antibiotics were tested in the panels: amikacin, aztreonam, cefepime, ceftazidime, ciprofloxacin, doripenem, gentamicin, imipenem, levofloxacin, meropenem, piperacillin/tazobactam, piperacillin, and tobramycin. MICs were analyzed and interpreted according to the recommended clinical breakpoints given in CLSI guidelines [21]. ATCC Quality control organisms used were *P. aeruginosa* ATCC 27853 and *Escherichia coli* ATCC 25922. Nonsusceptibility includes a combination of resistance and intermediate resistance. Multidrug (MDR) *P. aeruginosa* was defined as nonsusceptibility to at least one agent in three or more antimicrobial categories according to Magiorakos et al. [22]. Multiple antibiotic resistance index (MARI) was calculated and interpreted for the isolates as described by Gufe et al. [23]. Briefly, it is described as the ratio of the number of antibiotics to which isolates were resistant (a), to the total number of antibiotics to which the isolates were exposed (b), that is, multiple antibiotic resistance index (MARI) = *a*/b. Bacteria having MARI >0.2 originate from high-risk sources of contamination where several antibiotics have been used, while MARI value ≤ 0.2 indicates strains from sources where antibiotics have seldom or never been used.

### 2.7. Molecular ESBL and MBL Detection by rPCR

Isolated *P. aeruginosa* colonies on Cetrimide agar and CHROMagar *Pseudomonas* were selected for genomic DNA extraction. Fifteen multidrug isolates were selected from the pool using a simple random sampling technique. Template DNA was extracted by MagNA Pure Compact (MPC) using MPC Nucleic Acid isolation kit according to the manufacturer's instruction. Real-time PCR was carried out in the LightCycler 2.0 instrument (Roche Applied Science, Germany) using Fast start LightCycler 480 HybProbes Master Kit (Roche Diagnostics, USA). Specific primers and probes (Table 3) targeting the genes CTX-M, SHV, TEM, IMP, and VIM were amplified by singleplex rPCR using the same protocol described in Table 2. Primers were designed by TIB Molbiol (Berlin, Germany). rPCR assay was performed in 32 carousels using 20 *μ*L capillaries with each capillary containing a total volume of 20 *μ*L, including 2 *μ*L of LightCycler FastStart DNA Master Hybridization Probes (Roche Diagnostics), 2 *μ*L of primers (0.5 mM for each forward and reverse), 2 *μ*L of the probe, 2.4 *μ*L of MgCl2, 2 *μ*L of extracted DNA, and water to make up the volume of 20 *μ*L.

Absolute quantification was carried out using the LightCycler software 4.05. Data were obtained during the annealing period. Fluorescence was measured once every cycle immediately after the 60°C incubation (extension step). Fluorescence curves were analyzed with the LightCycler software, version 4.05. Results were expressed by determination of threshold cycle (Ct) value, which signified the cycle at which sample fluorescence became remarkably different from the baseline signal. Positive control strains used included *Klebsiella pneumoniae* ATCC 51503 (*bla*_CTX-M_), *Klebsiella pneumoniae* ATCC 700603 (*bla*_SHV_), *Escherichia coli* NCTC 13351 (*bla*_TEM_), *P. aeruginosa* NCTC 13437 (*bla*_VIM_), and *Escherichia coli* NCTC 13476 (*bla*_IMP_). These were obtained from the National Institute of Communicable Diseases (NICD), Johannesburg, South Africa.

### 2.8. Statistical Analysis

All the data was entered into an Excel sheet and uploaded onto the SPSS software (version 23.0 IBM, Armonk, NY). The prevalence of multidrug-resistant (MDR) *P. aeruginosa* and their distribution from different sources (water and abattoir wastewater) were determined and expressed as percentages.

## 3. Results

### 3.1. Isolation and Antimicrobial Susceptibility Testing

**Figure 1:**
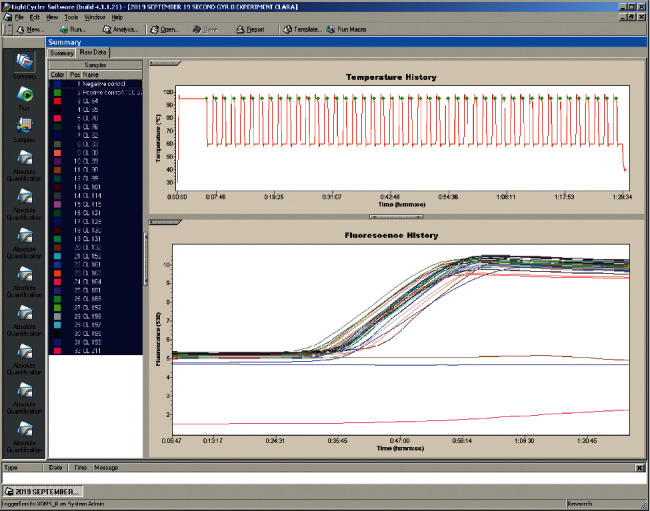
Amplification of gyr B (real time with LightCycler 2.0) in *P. aeruginosa* strains including reference strain ATCC 27853.

**Figure 2:**
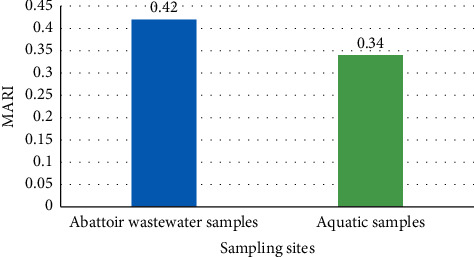
Mean MARI values in abattoir wastewater and aquatic *P. aeruginosa* isolates.

During the period of study, fifty-one isolates of *Pseudomonas* species were recovered, out of which thirty-six isolates were *P. aeruginosa* (70.6%) and fifteen were *P. fluorescens/putida* (29.4%). *P. aeruginosa* was the predominant species, of which nineteen (52.8%) and seventeen (47.2%) originated from surface water and abattoir wastewater, respectively. Of these, the 36 strains of *P. aeruginosa* were selected for further confirmation. They were confirmed by the real-time amplification of the *gyr*B gene including the reference strain, ATCC 27853 (Figure 1). The results of antibiotic susceptibility testing of *P. aeruginosa* strains showed varying levels of resistance. Of the clinically relevant antibiotics in the panel, there was resistance to aztreonam (86.1%), ceftazidime (63.9%), piperacillin (58.3%), cefepime (55.6%), imipenem (50%), piperacillin/tazobactam (47.2%), meropenem (41.7%), and levofloxacin (30.6%) (Table 4). Twenty out of thirty-six *P. aeruginosa* strains presented multidrug resistance profiles and were classified as MDR (55.6%) with 60% of the MDR strains originating from abattoir wastewater and 40% being from surface water. Most of the bacteria isolates showed a high MARI ranging from 0.08 to 0.69 with a mean MARI of 0.38. The mean MARI of isolates from abattoir wastewater was 0.42 while that of aquatic samples equals 0.34 (Figure 2).

### 3.2. Molecular Detection of Extended Spectrum *β*-Lactamase (ESBL) and Metallo-*β*-Lactamase (MBL) Encoding Genes in *P. aeruginosa*

PCR screening of genes encoding ESBL and MBL indicated the amplification of *bla*_SHV_, *bla*_CTX-M_, and *bla*_TEM_ in some of the *P. aeruginosa* isolates. Results of molecular detection of ESBL and MBL genotypes in environmental strains of *P. aeruginosa* by rPCR are presented in Table 5. Among the 15 *P*. *aeruginosa* isolates analyzed, 14 isolates (93.3%) harbored the *bla*_SHV,_ six isolates (40%) harbored the *bla*_TEM_, and three isolates (20%) harbored *bla*_CTX-M_. Only one isolate (6.7%) harbored the *bla*_VIM_ gene while no isolate was detected harboring the MBL, *bla*_IMP_.

## 4. Discussion

In this study, thirty-six isolates of *P. aeruginosa* were recovered from abattoir wastewater and surface water. In agreement with this study, Igbinosa et al. [27–29] have all reported the occurrence of *P. aeruginosa* from hospital drains, environmental, and wastewater networks from various parts of the world. The occurrence of this microorganism is a cause of concern given that it is an opportunistic human pathogen and can infect people whose immunity is compromised [30]. A similar study in Nigeria found that the discharge of effluents from abattoir directly into water bodies without prior treatment has triggered serious health risks subsequent to its contamination by bacteria [14].

The prevalence rate of *P. aeruginosa* (70.6%) seen in the current study is comparable to previous reports from Nigeria on water samples from fish pond sites and cattle waste, where *P. aeruginosa* was found to be the most prevalent with the highest occurrence rate of 62.8% and 71.5%, respectively, among other species [31, 32]. This possibly could be due to the physiological versatility and limited nutritional requirements that enable it to adapt in adverse conditions [33]. Likewise, in agreement with this study, a study carried out in Mafikeng in the North West Province of South Africa isolated *P. aeruginosa* from both drinking and surface waters [34]. However, contrary to our findings, a study carried out in Alice, Eastern Cape, South Africa, on wastewater samples found a lower occurrence rate of 11.1% [35]. This disparity is most likely due to different treatment processes used in water purification, or it can be assumed that wastewater treatment plant (WWTP) does not totally eliminate bacteria especially MDR strains since these organisms are resilient to the treatment processes and eventually play a role in the transmission and spread of antimicrobial resistance.

The resistance profiles of the isolates revealed 63.9% and 55.6% resistance to the second- and third-generation cephalosporin, respectively (ceftazidime and cefepime) and low resistance to aminoglycosides. The observed resistance pattern in cephalosporins observed in the current study is at par with the previous report of Ejikeugwu et al. [28] but lower compared to that of Tapela et al. [29, 36] with a 100% resistance rate. However, Benie et al. from Cote d'Ivoire [37] reported lower rates of resistance of 6.9% and 17% to ceftazidime and cefepime, respectively. A cause for concern is the high resistance displayed to the cephalosporins, which are frontline antipseudomonal drugs for treating *P. aeruginosa* infections; increased resistance to this class of antibiotics will not be favorable and will result in limited treatment options. The present study revealed that 16.7% of isolates were resistant to aminoglycoside, amikacin, and gentamicin. A similarly low rate of resistance to amikacin (19%) but slightly higher rate to gentamicin (28.5%) was reported by a study carried out in Egypt [36]. However, an elevated resistance rate of 79% in gentamicin has been reported by a study carried out in Nigeria [28]. This variation could be due to differences in the prescription pattern of aminoglycoside antibiotics [38].

Resistance to carbapenems including imipenem (50%) and meropenem (41.7%) was also observed in the current study. This is quite unexpected, given the fact that carbapenems represent one of the most effective and among the best options for treating Gram-negative infections particularly MDR infections. Carbapenem-resistant *P. aeruginosa* isolates are frequently associated with a higher mortality rate due to the enzyme carbapenemase mediating the resistance and a higher likelihood of extensive spread of resistance through mobile genetic elements [39]. The abuse of antimicrobials in human and veterinary medicine often leads to a proliferation of antibiotic-resistant bacteria (ARB) and antibiotic-resistant genes (ARGs) that can be transferred to human pathogenic bacteria. This transfer eventually nullifies the efficacy of current and upcoming antibiotics, thereby leading to treatment failure for some life-threatening diseases [40].

In the current study, the prevalence of 55.6% MDR *P. aeruginosa* (MDRPA) in a nonclinical setting is high and alarming. Consequently, this study proved the presence of MDR *P. aeruginosa* in the nonclinical environment in the Eastern Cape Province of South Africa. This finding is consistent with the report of Algammal et al. [41] with 55.5% MDR strains of *P. aeruginosa* from freshwater fish samples. However, Olga et al. [42] reported a lower rate of MDRPA of 32% from the aquatic environment. The irrational and unwarranted use of antibiotics fast-tracks the increase of multidrug-resistant strains, thus rendering empirical antibiotic therapy ineffective [43].

MAR indexing method is a simplified rapid method of distinguishing organisms from different origins either from high-risk sources of contamination where antibiotics are frequently used or from low-risk sources [44] and as an indicator of the level of contamination potentially unsafe for humans [45]. A MAR index >0.2 indicates that isolates originate from high-risk sources of contamination [46]. In the current study, the analysis of the MAR index of the *P. aeruginosa* strains showed that all of them had a MAR index above 0.2 (Figure 2). Odjajare et al. [47] and Gufe et al. [23] reported similar results. The findings reflect the overuse of antibiotics in animal production and highlight the sources of these pathogens, which eventually translocate into water bodies and pose health risks to humans.

The release of MDR bacteria including ESBL and MBL producers into water bodies is a cause of concern. These organisms could act as opportunistic pathogens when they persist in the environment, and since they carry mobile genetic material, they can serve as a resistance pool that could fast-track the spread of antimicrobial resistance [48]. In the current study, *bla*_SHV_ was the most prevalent ESBL detected by PCR. This was detected in 14 isolates (93.3%). *bla*_TEM_ was detected in 40% of the isolates while the least detected ESBL was *bla*_CTX-M_ (20%). This study is in agreement with other authors [49, 50]. Together, these statistics suggest successful dissemination of the ESBL-encoding genes universally.

### 4.1. Limitations

The limitation of the study is that the study was based on a small sampling size.

## 5. Conclusions

The findings of this study revealed a considerable burden of resistance against important antibiotics such as ceftazidime, cefepime, imipenem, and meropenem including piperacillin and piperacillin/tazobactam, which are antibiotics of choice for treating MDR *P. aeruginosa*. This poses complications to the successful treatment of human infections. Given the public health relevance, the results of this study reveal the importance and necessity of concerted surveillance of antimicrobial resistance and resistance genes in the nonclinical environment at both local and regional levels and the implementation of the One Health approach. In addition, the occurrence of ESBL-producing *P. aeruginosa* presents a potential public health threat since the genetic elements responsible for this resistance are present on mobile genetic elements (MGEs) that can be transferred to other Gram-negative bacteria through horizontal gene transfer.

## Figures and Tables

**Table 1:** Primer sequences for detection of *gyr*B genes.

Primers	Primers sequences (5′-3′)	Tm in 0°C	References
gyr B forward primer	CCT GAC CAT CCG TCG CCA CAA	55.3	[19]
Gyr B reverse primer	CGC AGC AGG ATG CCG ACG CC	53.1	
Gyr B probe 1	FAM-CCG TGG TGG TAG ACC TGT TCC CAG ACC-BHQ		This study
Gyr B probe 2	FAM-CCG TGG TGG TAG ACC TGT TCC CAG ACC-BBQ		

**Table 2:** rPCR cycle protocol.

Protocol	Temperature (°C)	Acquisition mode	Time	Ramp rate	Cycle
Denaturation	95	None	5 minutes	4.4	1
Quantification: Annealing	95	None	30 seconds	4.4	45
Extension	60	Single	1 minute	4.4	
Cooling	40	None	30 seconds	4.4	1

**Table 3:** Primer sequences for detection of *bla*_CTX-M_, *bla*_SHV_, *bla*_TEM_, *bla*_IMP_, and *bla*_VIM_ genes.

Primers	Primers sequences (5′-3′)	Tm in 0°C	References
CTX-M forward primer	ATGAGYACCAGTAARGTKATGGC	58.7	[24]
CTX-M reverse primer	ATCACKCGGRTCGCCIGGRAT	59.3	
CTX-M probe	FAM-CCCGACAGCTGGGAGACGAAACGT-BBQ	70.2	
SHV forward primer	TCCCATGATGAGCACCTTTAAA	56.8	[25]
SHV reverse primer	TCCTGCTGGCGATAGTGGAT	58.6	
SHV probe	FAM-TGCCGGTGACGAACAGCTGGAG-BBQ	68.3	
TEM forward primer	GCATCTTACGGATGGCATGA	56.6	[25]
TEM reverse primer	GTCCTCCGATCGTTGTCAGAA	57.7	
TEM probe	FAM-CAGTGCTGCCATAACCATGAGTGA-BHQ1	62.2	
IMP forward primer	GGGCGGAATAGAGTGGCTTA	57.6	[26]
IMP reverse primer	GGCTTGAACCTTACCGTCTTTTT	59.3	
IMP probe	FAM-CGATCTATCCCCACGTATGCATCTGAATTAACA-BHQ1	67.4	
VIM forward primer	TGCGCTTCGGTCCAGTAGA	59.0	[26]
VIM reverse primer	TGACGGGACGTATACAACCAGAT	58.5	
VIM probe	FAM-CTTCTATCCTGGTGCTGCGCATTCG-BHQ1	67.6	

**Table 4:** Antibiotic resistance pattern of *P. aeruginosa* isolates.

Antibiotic	No (%) resistant	No (%) susceptible
Amikacin	6 (16.7)	30 (83.3)
Aztreonam	31 (86.1)	5 (13.9)
Ceftazidime	20 (55.6)	16 (44.4)
Cefepime	23 (63.9)	13 (36.1)
Ciprofloxacin	8 (22.2)	28 (77.8)
Doripenem	5 (13.9)	31 (86.1)
Gentamicin	6 (16.7)	30 (83.3)
Imipenem	18 (50)	18 (50)
Levofloxacin	11 (30.6)	25 (69.4)
Meropenem	15 (41.7)	21 (58.3)
Piperacillin	21 (58.3)	15 (41.7)
Piperacillin/tazobactam	17 (47.2)	19 (52.8)
Tobramycin	3 (8.3)	33 (91.7)

**Table 5:** Extended-spectrum *β*-lactamase (ESBL) and metallo-*β*-lactamase (MBL) gene types detected in nonclinical isolates of *P. aeruginosa*.

Positive by PCR for ESBL genes	Number amplified (*N* = 15)	Total (%)
A. Single ESBL gene		
*bla*_SHV_	14	93.3
*bla*_TEM_	6	40.0
*bla*_CTX-M_	3	20.0
*bla*_VIM_	1	6.7
*bla*_IMP_	0	0
B. Two or more ESBL genes		
*bla*_TEM_ + *bla*_SHV_	6	40
*bla*_TEM_ + *bla*_CTX-M_	3	20
*bla*_SHV_ + *bla*_CTX-M_	3	20
*bla*_SHV_ + *bla*_VIM_	1	6.7

## Data Availability

All data generated or analyzed during this study are included in this published article.
